# Effect of 5E rehabilitation management model on physical and psychological health of elderly patients with cerebral infarction: a randomized controlled trial

**DOI:** 10.1186/s12912-026-04671-3

**Published:** 2026-04-24

**Authors:** Xiao Qin, Xueyan Cui, Wei Li, Jinhua Zhang, Yan Yuan, Haiyan Zhang, Hongxia Zhang, Chenke Wang, Haixu Ji

**Affiliations:** 1 Department of Nursing, North Henan Medical University, Henan, China; 2 Department of Nursing, Henan Medical University, Henan, China; 3 Department of Neurology, The First Affiliated Hospital of Henan Medical University, Henan, China

**Keywords:** Aging, Stroke, Nursing, Rehabilitation nursing, Motor function

## Abstract

**Background:**

Disability resulting from cerebral infarction (CI) persists throughout the lives of elderly survivors, severely impairing both their physical and mental health and diminishing their quality of life. However, current interventions have shown limited effectiveness in improving the quality of life of stroke patients, and the impact of the 5E rehabilitation management model on their physical and mental health remains unclear.

**Objective:**

This study aimed to apply the 5E rehabilitation management model to elderly patients with CI and evaluate its physical and mental impact on patients.

**Methods:**

A total of 84 elderly patients with CI who were hospitalized at The First Affilicated Hospital of Xinxiang Medical University were selected as the research subjects and randomly divided into an intervention group and a control group, with 42 patients in each. The 5E rehabilitation management model was applied to the intervention group (*n* = 42) as an early rehabilitation programme for elderly patients with CI. Routine nursing was employed for the control group (*n* = 42). Before the intervention, 1 month and 2 months after the intervention, patients in both groups were evaluated using the Manual muscle strength examination grading, the Brunnstrom motor function evaluation, the Modified Barthel Index, the PHQ-9, and the GAD-7. This study adhered to the CONSORT 2010 guidelines.

**Results:**

During follow-up, the 5E intervention group showed significantly greater improvement than the control group in muscle strength (upper limb: 3.79 ± 0.42 vs. 3.02 ± 0.64 at 2 months, *p* < 0.001), motor function, daily living ability (MBI: 71.45 ± 10.31 vs. 59.57 ± 9.91, *p* < 0.001), and reduced anxiety (7.38 ± 1.53 vs. 9.52 ± 2.51, *p* < 0.001) and depression (6.76 ± 1.21 vs. 8.90 ± 2.32, *p* < 0.001), with the effects being more pronounced at the 2-month follow-up.

**Conclusion:**

The 5E rehabilitation management model significantly improves muscle strength, motor function, daily living ability, and psychological health in elderly patients with CI, with effects becoming more pronounced over time. Multi-center studies with longer follow-up are warranted to confirm long-term efficacy.

**Trial registration:**

Chinese Clinical Trial Registry (ChiCTR2300070052). Recruitment: January to September 2022. [Registration date 2023/3/21. (Retrospectively registered)].

**Supplementary Information:**

The online version contains supplementary material available at 10.1186/s12912-026-04671-3.

## Background

Stroke is a global health issue and remains the second leading cause of death and disability worldwide, affecting over 80 million survivors [1, 2]. Cerebral infarction (CI), a common type of ischemic stroke, results from impaired cerebral blood flow leading to ischemia, hypoxia, and subsequent necrosis or softening of brain tissue [3]. In China, the incidence of CI continues to rise due to the aging population [4]. Approximately 2.5 million individuals experience their first CI annually, of whom nearly two-thirds are elderly adults [5]. The morbidity and mortality associated with CI remain high [6]. Limb dysfunction is one of the most prevalent and debilitating sequelae among elderly patients with CI, substantially diminishing their quality of life [7, 8]. Moreover, CI not only impairs patients’ self-care ability and motor function but also adversely affects their mental health, placing a significant burden on families and society due to disability and long-term care needs [9, 10].

Recent evidence underscores the importance of early rehabilitation for patients following CI, highlighting its role in controlling disease progression and promoting functional recovery [11, 12]. Chinese clinical guidelines recommend initiating passive and active exercise as soon as the patient’s condition stabilizes [13]. The World Health Organization (WHO) advises commencing rehabilitation within 48 h of stabilization of vital signs [14]. Studies have demonstrated that starting rehabilitation training within two weeks after CI onset can effectively reduce short-term mortality and enhance motor function recovery [15, 16]. However, further research is needed to determine whether early rehabilitation nursing intervention initiated within this two-week window yields optimal outcomes.

Early nursing intervention has been shown to facilitate neurological recovery, reduce complications, and improve quality of life in patients with CI [17]. For instance, combining early rehabilitation with virtual reality (VR) training has been found to enhance muscle strength, mood, and functional status [18]. Similarly, early rehabilitation nursing integrated with finger exercises effectively improves neurological, cognitive, and motor functions in patients with CI [19].

Patients with CI are also at heightened risk for negative psychological outcomes, including anxiety and depression [20]. Nearly 30% of post-stroke patients experience depression, which may hinder motor recovery and negatively impact quality of life and rehabilitation outcomes [21–23]. Patients are particularly vulnerable to psychological distress during the acute, unstable phase of the disease, prompting nurses to increasingly address mental health needs [24, 25]. Wang et al. [26] reported that combining virtual reality technology with conventional rehabilitation significantly alleviated anxiety and depression while improving quality of life in patients with CI.

The 5E rehabilitation management model, proposed by the International Rehabilitation Association in 1994, comprises five core components: encouragement, education, exercise, employment, and evaluation [27]. Encouragement involves psychological support to boost patient confidence and alleviate negative emotions. Education provides systematic health instruction to enhance disease-related knowledge. Exercise consists of standardized rehabilitation training programs aimed at improving motor function and quality of life. Employment refers to activities that enhance patients’ ability to perform daily tasks and promote self-care. Evaluation entails ongoing assessment of nursing interventions, analysis of suboptimal outcomes, and iterative refinement of the care plan. This multidimensional model emphasizes the nurse’s role in addressing patients’ physiological, psychological, and functional needs, making it potentially well-suited for the rehabilitation of elderly patients with CI.

The 5E model has been widely applied in chronic disease management with promising results. In patients with chronic obstructive pulmonary disease (COPD), the 5E-based rehabilitation nursing model improved self-management behaviors [28]. Among peritoneal dialysis patients, it enhanced self-management capacity, ameliorated malnutrition and micro-inflammatory status, and slowed the progression of residual renal dysfunction [29, 30]. Additionally, an information-based 5E rehabilitation management program significantly improved medication adherence and quality of life in patients with aortic dissection complicated by obstructive sleep apnea [31].

In summary, while early rehabilitation benefits patients with cerebral infarction (CI), existing interventions are often limited to single components and lack systematic structure and clear specifications [11–16]. The 5E rehabilitation management model, effective in other chronic conditions [28–31], provides a multidimensional framework integrating psychological support, health education, structured exercise, daily living training, and ongoing evaluation. A study by Nian et al. [32] reported that combining the 5E model with continuity of care improved outcomes in acute ischemic stroke patients; however, that study was retrospective, did not focus on elderly patients, and was not a randomized trial of the standalone nurse-led 5E model. To date, no prospective randomized controlled trial has systematically applied the 5E model specifically in elderly patients with CI.

Therefore, this study was designed to develop and evaluate a structured nurse-led 5E rehabilitation intervention for this population. Unlike previous studies focusing on isolated components or intervention timing, this study adopts a comprehensive multicomponent approach targeting muscle strength, motor function, activities of daily living, anxiety, and depression. The primary objective is to assess the effects of this model using a randomized controlled trial design, thereby providing an evidence-based rehabilitation protocol for clinical nursing practice in elderly patients with CI.

## Methods

### Intervention development

The 5E rehabilitation management model for elderly patients with CI was developed prior to the trial through a two-stage process: a literature search and a two-round Delphi expert consultation.

#### Literature search

A systematic search in Chinese and English databases (CNKI, Wanfang, VIP, CBM, PubMed, Web of Science, EMBASE, Cochrane) was conducted from inception to May 2023. Search terms included combinations of “cerebral infarction/ischemic stroke,” “elderly patients,” and “rehabilitation/nursing intervention.” After screening, 13 articles met the inclusion criteria. Based on the 5E model, an initial intervention draft was developed, comprising 5 first-level indicators (encouragement, education, exercise, employment, evaluation) and 16 s-level indicators.

#### Expert consultation

A two-round Delphi survey was conducted with 15 experts in neurology, rehabilitation medicine, and rehabilitation nursing (all held associate senior professional titles or above). In the first round, experts rated the importance of each indicator and provided suggestions for revision. Based on their feedback, the intervention was refined by adding “case sharing” under encouragement, “individualized guidance” under education, “active exercise”under exercise, and “pre-intervention evaluation” under evaluation. The second round confirmed consensus. Expert authority coefficient was 0.89, Kendall’s coefficients of concordance were 0.148 and 0.143 (both *p* < 0.05), and all indicators met the prespecified criteria (mean importance score > 4.0 and coefficient of variation < 0.25). The final intervention framework comprised 5 first-level indicators and 20 second-level indicators, as detailed in Table 1. This finalized protocol was fixed prior to participant enrollment and used throughout the trial.

**Table 1:** 5E Rehabilitation management model for elderly patients with CI

First-level indicator	Second-level indicator		Contents	Frequency
Encourage	Encourage expression		Encourage patients to express their inner thoughts and help them at any time according to their psychological condition	Once a day
	Case sharing		Share successful cases of treatment with patients and encourage patients to actively cooperate with treatment	During hospitalization
	Peer encouragement		Guide patients in the same ward to encourage each other	On admission
	Family Communication Guidance		Guide family members in mastering basic communication skills, striving to achieve patient listening, active responsiveness, and effective management of sudden emotional outbursts.	Once a week
	Follow-up after discharge		Follow up on phone or WeChat after discharge to understand physical and mental conditions and provide timely supervision and guidance	Once a week
Education	Collective health education		Group sessions (3–5 patients and their caregivers) covering: causes of CI, risk factors, importance of rehabilitation, medication adherence, diet, and fall prevention. Use PowerPoint slides and printed leaflets.	During the period of hospitalization, 3 times, 30–40 min per session
	individualized guidance		Ascertain level of patient’s knowledge of health issues by asking questions, and yet giving guidance to patients with health problems	Once a week
	Follow-up after discharge		Following discharge, contact patients by phone or WeChat to encourage them to keep a healthy lifestyle, pay attention to diet, control their emotions, take medicine rigorously according to the doctor’s advice and answer their questions	Once a week
Exercise	Turn over		Turn over the patient regularly	Every 2 h
	Good limb position placement		According to the condition, place patients in the supine position, healthy lateral position, patient lateral position and bed sitting position	Every 2 h
	Passive movement of upper limbs		Include shoulder joint motion, elbow joint motion and wrist joint motion	During the period of hospitalization, once a day, every 20–30 min
	Passive hand movement		Include metacarpophalangeal joint motion and interphalangeal joint motion	
	Passive movement of lower limbs		Include hip and knee joint mobility, as well as ankle and foot joint movement	
	Active movement		According to the patient’s condition, increase the range of exercise gradually, step by step. Use Bobath handshake for upper limb rehabilitation training, for lower limb rehabilitation training, use bridge exercise, sitting up and sitting balance training, as well as standing up and standing balance training and walking training	Once a day for 20–30 min
	Body position transfer		Including turning over in bed, moving in bed position, transfer between lying position and sitting position, transfer between sitting position and standing position, bed-chair transfer, etc.	Once a day
		Follow-up after discharge	Post-discharge follow-up via phone or WeChat, along with video playback guidance for exercises, enables timely monitoring of rehabilitation progress, addresses questions, and provides personalized recommendations. Family members are encouraged to participate in rehabilitation support.	Once a week
Employment		Daily living ability training	Train patients in daily life skills and encourage them to work hard on activities such as washing their faces and eating	Once a day
		after discharge	Phone or WeChat follow-up after discharge to keep abreast of daily life and provide personalized guidance and advice	Once a week
Evaluation		Admission evaluation	Investigate the basic information and the comorbidity situation of patients	On admission
		Pre-intervention evaluation	Start the intervention 24 h after the patient is stabilized; before the intervention, assess the patient’s mental mastery, physical rehabilitation exercise, daily ability exercise, etc., and give feedback on the results	Before intervention
		Evaluation of intervention effect	Evaluate the rehabilitation effect on patients	After intervention

Note: CI, cerebral infarction. The intervention began 24 h after the patient’s condition stabilized and was individualized based on the patient’s condition and self-care ability

### Study design

This was a single-blind, parallel-group, single-center randomized controlled trial conducted in accordance with the CONSORT guidelines. Ethical approval was obtained from the Xinxiang Medical University Institutional Review Board (Approval no. XYLL-2020250). This trial was registered in the China Clinical Trial Registry on March 21, 2023 (ChiCTR2300070052). [Retrospectively registered].

### Randomization and allocation

The random sequence was generated using a computerized block randomization method (block size = 4) via SPSS 25.0 software by an independent statistician who was not involved in participant recruitment or intervention delivery. Group assignments (intervention group vs. control group) were placed in sequentially numbered, opaque, sealed envelopes, which were prepared by a research assistant not involved in enrollment or data collection. A trained research nurse, blinded to the allocation sequence, performed participant recruitment and eligibility assessment. After obtaining informed consent, the recruiting nurse opened the next sequentially numbered envelope to reveal the participant’s group allocation. To minimize the risk of contamination, participants in the intervention and control groups were placed in different hospital wards (the second and third floors, respectively). Although this measure was intended to reduce interaction between groups, we acknowledge that it may have introduced potential bias due to differential environmental influences. This limitation is discussed further in the Discussion section.

### Blinding

Due to the nature of the intervention, neither participants nor intervention providers could be blinded to group allocation. However, outcome assessors and data analysts were blinded to group assignments throughout the study to reduce detection and analysis bias.

### Participants and sample size

From January to September 2022, the First Affiliated Hospital of Xinxiang Medical University consecutively recruited 84 hospitalized elderly patients with cerebral infarction as study subjects, constituting a convenience sample. The inclusion criteria were: (a) meeting the diagnostic criteria of the *China Guidelines for the Diagnosis and Treatment of Acute Cerebral Infarction 2014*; (b) aged 65 years or older; (c) first-ever clinically confirmed CI based on medical history and neuroimaging (CT or MRI), with no prior history of stroke; (d) presence of hemiplegia symptoms after stroke onset; (e) clear consciousness; and (f) provision of written informed consent. For patients with uncertain symptom onset time (e.g., wake-up strokes), inclusion required that the time of onset could be estimated within 24 h based on the last-known-well time, in accordance with stroke management guidelines.

The exclusion criteria were: (a) critical illness; (b) limb dysfunction caused by other conditions (e.g., arthritis, femoral head necrosis); (c) major comorbidities (e.g., heart, liver, or kidney disease); (d) participation in other clinical trials; and (e) refusal to participate in post-discharge follow-up. Dropout criteria included: (a) voluntary discharge from the hospital; (b) loss to follow-up after discharge; and (c) voluntary withdrawal during the study. Participants who developed other acute diseases during the study period were also excluded.

Sample size was calculated using G*Power version 3.1 [33]. For repeated-measures ANOVA with two groups and three measurements, assuming a significance level of α = 0.05, power of 0.80, correlation among repeated measures of 0.50, and a medium effect size of f = 0.25, the required sample size was approximately 29 participants per group. To account for an anticipated dropout rate of 10%, the target sample size was increased to 32 per group, resulting in a total of 64 participants.The effect size f = 0.25 was selected based on previous meta-analyses reporting large effect sizes for early rehabilitation on daily living ability (d = 0.97) and anxiety reduction (d = 0.93) in stroke populations [34, 35]; a medium effect size was chosen as a conservative estimate for sample size calculation according to conventional criteria [36].To further ensure adequate power and account for potential attrition, a total of 84 participants were enrolled in this study.

### Intervention

#### Intervention group

Participants in the intervention group received a structured 2-month rehabilitation program based on the 5E management model (encouragement, education, exercise, employment, evaluation). The detailed content and frequency of each component are presented in Table 1. A more comprehensive description of the intervention, including session-by-session protocols, educational materials, and standardized scripts for telephone follow-ups, is provided in Supplementary File 1. The intervention was delivered by four research nurses who had undergone standardized training. Prior to the trial, all four nurses completed a 2-week training program conducted by a senior rehabilitation specialist and a neurologist. The training covered: (1) the theoretical framework of the 5E model; (2) hands-on practice of passive and active rehabilitation exercises; (3) assessment methods for muscle strength (MMT) and motor function (Brunnstrom stages); and (4) communication skills in providing psychological support and health education. Within this structured program, exercise routines and activities of daily living training were tailored to each patient’s specific condition, stage of recovery, and self-care capabilities. A competency assessment was conducted at the end of the training, and all nurses achieved a passing score (> 85%) before participating in the study.

The intervention began 24 h after the patient’s vital signs and neurological symptoms had stabilized. During hospitalization, all intervention sessions were delivered face-to-face in the patient’s ward. Each session was documented in a standardized intervention log, which recorded the date, duration, specific activities performed, and any deviations from the protocol. To ensure intervention fidelity, 20% of the sessions were randomly monitored by a senior nurse supervisor who was not involved in intervention delivery. Feedback was provided to the intervention nurses on a weekly basis. After discharge, participants received weekly telephone follow-ups (15–20 min each) to monitor adherence, answer questions, and provide encouragement. During these calls, participants were asked to report their home-based exercise activities, which were cross-referenced with exercise diaries maintained by patients or their caregivers. All follow-up calls were recorded and reviewed by the research team to ensure consistency in intervention delivery. In the intervention group, 100% of scheduled telephone follow-ups were completed, and 85% of patients submitted exercise diaries.

#### Control group

Participants in the control group received standard care during hospitalization and after discharge. This included a comprehensive health education session covering the etiology, clinical manifestations, treatment options, and prognosis of stroke, as well as guidance on dietary modifications, smoking and alcohol cessation, and appropriate physical activity. During hospitalization, if participants reported any symptoms, the nursing staff promptly notified the physician, implemented the corresponding medical orders, and provided routine health guidance. At discharge, patients received advice on post-discharge self-management and were reminded of their scheduled follow-up visits.

#### Outcomes

Outcomes were measured three times: the day before intervention (T0), 1 month post-intervention (T1) and 2 months post-intervention (T2).

The following instruments were selected based on their widespread use in stroke rehabilitation research, established reliability and validity in elderly stroke populations, and sensitivity to change over short periods. Manual muscle strength examination grading (also known as Manual Muscle Testing, MMT) and Brunnstrom motor function evaluation were used to assess muscle strength and motor recovery, respectively. The Modified Barthel Index (MBI) was employed to evaluate functional independence. Depressive and anxiety symptoms were measured using the Patient Health Questionnaire-9 (PHQ-9) and the Generalized Anxiety Disorder 7-item Scale (GAD-7), both of which are brief, validated screening tools for this population.

#### Primary outcomes

##### Manual muscle strength examination grading

Manual Muscle Testing (MMT) was used to assess the muscle strength of patients’ upper and lower limbs [37]. Muscle strength was graded on a scale of 0 to 5 according to the following criteria: Grade 0: No muscle contraction detected; Grade 1: Faint contraction, but no joint movement; Grade 2: Full range of joint movement with gravity eliminated; Grade 3: Full range of joint movement against gravity, but not against resistance; Grade 4: Full range of joint movement against gravity and some resistance; Grade 5: Full range of joint movement against gravity and full resistance. Each grade corresponds to a score from 0 to 5. MMT is performed by observing the range of active limb movement and palpating muscle contraction strength, relying on the examiner’s clinical judgment in accordance with standardized criteria.

##### Brunnstrom motor function evaluation

Motor function of the upper limbs, hands, and lower limbs was assessed using the Brunnstrom stages of motor recovery [38]. The stages are defined as follows: Grade I: Flaccidity; no voluntary movement. Grade II: Spasticity appears; basic limb synergies or some components may appear as associated reactions; slight finger flexion may occur. Grade III: Spasticity increases markedly; voluntary movement within limb synergies is possible; hip and knee flexion can occur when sitting and standing; fingers can be flexed voluntarily but cannot be extended (hook grasp). Grade IV: Spasticity begins to decline; movements deviating from basic synergies become possible; hand can perform lateral grasp and release with thumb movement; fingers can extend through a small range. Grade V: Spasticity further decreases; more complex movement combinations emerge; hand can grasp cylindrical and spherical objects, though movement may be clumsy; all fingers can extend voluntarily, but range may vary. Grade VI: Spasticity largely disappears; isolated joint movements are performed smoothly; coordination approaches normal levels; hand can perform various grasp tasks and individual finger movements, though speed and dexterity may be slightly reduced compared to the unaffected side. Scores from 1 to 6 were assigned corresponding to each stage. The highest stage achieved by the patient was recorded.

#### Secondary outcomes

##### Modified barthel index

Activities of daily living were assessed using the Modified Barthel Index (MBI) [39] with the simplified Chinese version. The MBI comprises 10 items covering grooming, bathing, stair climbing, eating, toileting, dressing, bowel control, bladder control, bed-to-chair transfer, and walking. Each item is scored based on the level of independence, with different scoring ranges for different items: Grooming, bathing, and walking: 0, 1, 3, 4, or 5 points; Eating, toileting, dressing, bowel control, bladder control, and stair climbing: 0, 2, 5, 8, or 10 points; Bed-to-chair transfer: 0, 3, 8, 12, or 15 points. The total score ranges from 0 to 100, with higher scores indicating greater independence. Functional status was categorized as follows: 100 points = complete independence; ≥60 = mild dysfunction; 41–59 = moderate dysfunction; 21–40 = severe dysfunction; ≤20 = complete dependence. The MBI demonstrated good inter-rater and intra-rater reliability, with Kappa values ranging from 0.89 to 0.99.

##### Anxiety and depression

This study employed the Chinese version of the Patient Health Questionnaire-9 (PHQ-9) [40] to assess depressive symptoms and the Chinese version of the Generalized Anxiety Disorder-7 (GAD-7) [41] to evaluate anxiety symptoms. Both scales are widely used in stroke rehabilitation research and have been shown to be valid and sensitive measures in elderly populations [42, 43]. Participants rated the frequency of symptoms over the previous two weeks on a four-point Likert scale ranging from 0 (not at all) to 3 (nearly every day). The PHQ-9 total score ranges from 0 to 27, with higher scores indicating more severe depressive symptoms; a cutoff score of ≥ 10 indicates moderate to severe depression [40]. The GAD-7 total score ranges from 0 to 21, with scores of 5, 10, and 15 representing mild, moderate, and severe anxiety, respectively [41]. In the present study, both scales demonstrated satisfactory internal consistency, with Cronbach’s α coefficients of 0.86 for the PHQ-9 and 0.88 for the GAD-7.

#### Study hypothesis

The following hypotheses were formulated to guide this study:

1. The 5E rehabilitation management model will lead to greater improvements in physical and psychological outcomes compared with usual care in elderly patients with cerebral infarction.
2. Patients in the intervention group will demonstrate significantly greater improvement in upper and lower limb muscle strength than those in the control group.
3. The intervention group will show superior recovery in motor function (upper limbs, hands, and lower limbs) compared with the control group.
4. The intervention group will exhibit greater enhancement in activities of daily living, as measured by the Modified Barthel Index, relative to the control group.
5. The intervention group will experience greater reductions in anxiety and depression symptoms, as assessed by the GAD-7 and PHQ-9, respectively, compared with the control group.

### Data collection

Data were collected by four trained graduate students under the supervision of the principal investigator. Baseline assessments were conducted in the hospital prior to the intervention. Follow-up assessments at one and two months post-intervention were conducted during home visits, which were scheduled in advance with participants and their family members. Muscle strength was assessed using MMT, motor function was evaluated using the Brunnstrom stages, and activities of daily living were measured using the Modified Barthel Index (MBI). Anxiety and depressive symptoms were assessed using the GAD-7 and PHQ-9, respectively. All questionnaires were self-administered by participants whenever possible. For participants unable to complete the questionnaires independently due to visual or motor impairments, a trained interviewer read the items aloud and recorded the responses using standardized prompts, strictly following the instruments’ guidelines and avoiding any interpretation or leading cues. All four graduate students used standardized instructions to explain the study procedures to participants and their families, ensuring consistency across all assessments.

### Data analysis

Data were analyzed using SPSS version 25.0 (IBM Corp., Armonk, NY, USA). Double-entry verification ensured data accuracy. Data were analyzed based on complete-case analysis, as only one participant was lost to follow-up. Given the low rate of loss to follow-up (1.2%), complete-case analysis was considered appropriate. A sensitivity analysis comparing the complete-case results with those obtained by imputing the missing data (using last observation carried forward) yielded consistent findings, supporting the robustness of the results. Descriptive statistics (mean ± SD for continuous variables; frequencies and percentages for categorical variables) summarized participant characteristics. Baseline comparability between the two groups was assessed using chi-square tests or Fisher’s exact tests for categorical variables, and independent samples t-tests for continuous variables. Normality of continuous outcomes was examined using the Shapiro-Wilk test and Q-Q plots, and homogeneity of variances was assessed using Levene’s test; all assumptions were satisfied for all outcomes. Mauchly’s test of sphericity was conducted for repeated-measures analyses. The assumption of sphericity was violated for anxiety (W = 0.05, *p* < 0.05) and depression (W = 0.02, *p* < 0.05); therefore, Greenhouse-Geisser corrections were applied for these outcomes. The assumption of sphericity was met for all other outcome measures.

Between-group differences at each time point (T0, T1, and T2) were examined using independent samples t-tests, with effect sizes reported as Cohen’s d, where 0.2, 0.5, and 0.8 indicate small, medium, and large effects, respectively [44]. To examine the effects of group, time, and their interaction, a two-way repeated-measures analysis of variance (ANOVA) was performed for each outcome measure. Partial eta-squared (η²) was reported as a measure of effect size, with values of 0.01, 0.06, and 0.14 representing small, medium, and large effects, respectively [45]. Due to the randomized design, no covariate adjustments were performed. All statistical tests were two-tailed, and *p* < 0.05 was considered statistically significant for the primary analyses.

### Ethical considerations

This study was conducted in accordance with the Declaration of Helsinki and approved by the Institutional Review Board of Xinxiang Medical University (Approval No. XYLL-2020250). All participants were informed of the study purpose and procedures, as well as the confidentiality of their data, prior to enrollment. Written informed consent was obtained from all participants before data collection.

### Quality control

Several measures were implemented to ensure data quality and minimize bias. Outcome assessors (four trained graduate students) were blinded to group allocation throughout the study. During home visits, they used standardized scripts and were instructed not to discuss the intervention with participants or their families. To prevent unblinding, assessors were trained to redirect any related questions to the research coordinator. Prior to the intervention, all personnel involved in intervention delivery received standardized training from a senior rehabilitation specialist, covering rehabilitation exercises, motor function assessment, muscle strength classification, and data collection procedures. The research team also included two neurologists, two physical therapists, and two rehabilitation nursing specialists who provided supervision throughout the study. When assessing motor function and muscle strength, a rehabilitation therapist provided on-site guidance or performed the assessment jointly with the researcher. The intervention group received weekly telephone follow-ups after discharge to monitor adherence and address questions. All data were double-entered and verified for accuracy, and statistical experts were consulted during analysis.

## Results

### Baseline characteristics

**Fig. 1:**
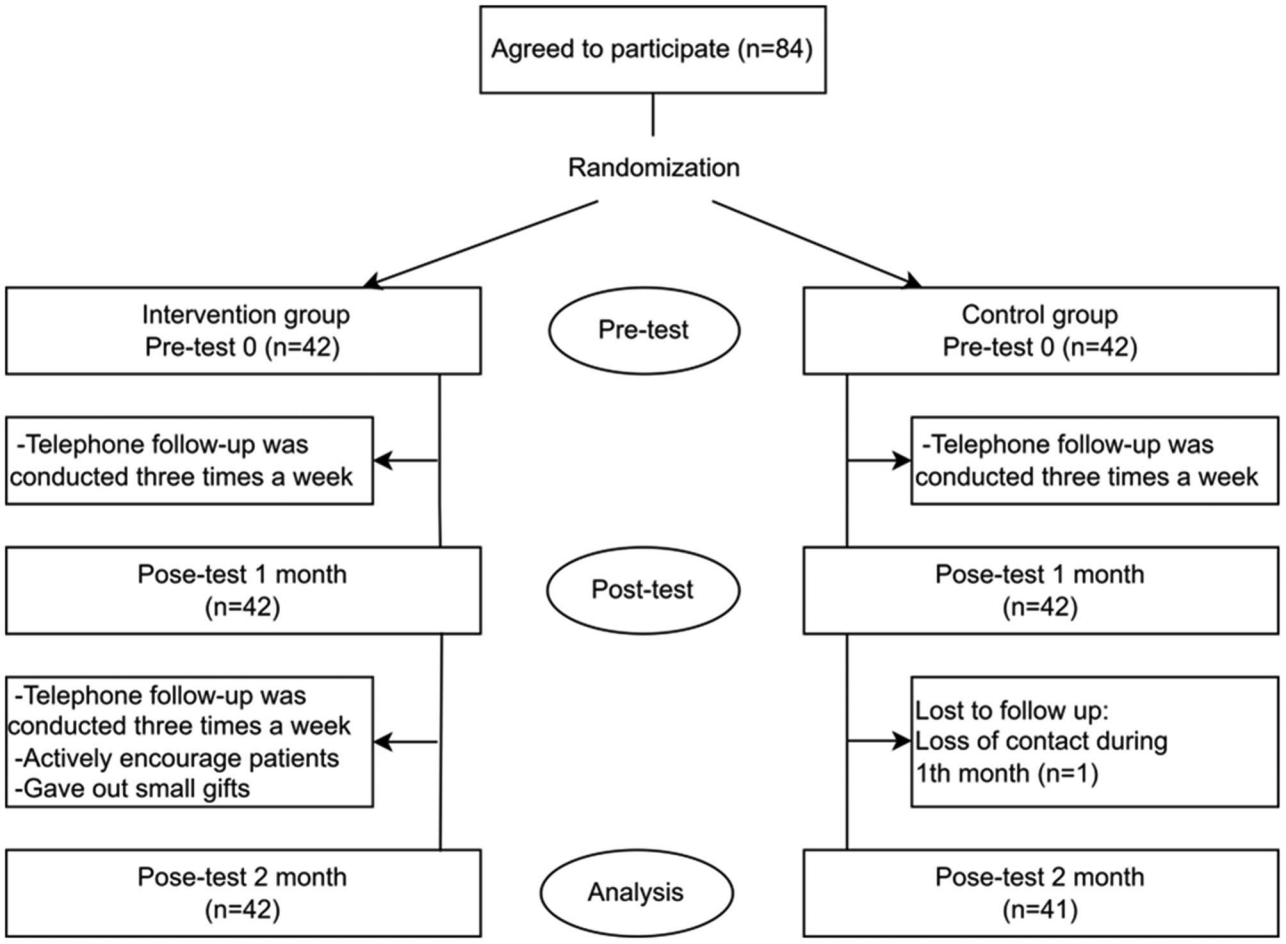
Flow diagram detailing the study design

Of the 84 enrolled participants, 83 completed the study, with one patient in the control group lost to follow-up. The intervention group comprised 42 participants, and the control group comprised 41 participants (Fig. 1). In both groups, most patients were aged 65–70 years (intervention group: 61.9%; control group: 54.8%). The most common comorbidities were hypertension (intervention group: 69.0%; control group: 71.4%) and heart disease (intervention group: 57.1%; control group: 52.4%). No significant differences in baseline characteristics were observed between the two groups (all *p* > 0.05; Table 2).

**Table 2:** Baseline demographic and clinical characteristics of participants (*N* = 83)

Variables	IG(*n* = 42) *n*(%)	CG(*n* = 41) *n*(%)	χ^2^/t	*p*
Gender, n (%)			0.049	0.825^a^
Female	18(42.9)	17(41.5)		
Male	24(57.1)	25(58.5)		
Age (years), n (%)			1.604	0.448^a^
65~	26(61.9)	22(53.7)		
70~	9(21.4)	14(34.1)		
80~	7(16.7)	5(12.2)		
Marital status, n (%)			0.309	0.578^a^
Married	35(83.3)	32(78.0)		
Widowed and Divorced	7(16.7)	9(22.0)		
Educational level, n (%)			4.618	0.202^a^
Illiteracy	7(16.7)	5(12.2)		
Primary school	16(38.1)	12(29.3)		
Junior high school	8(19.1)	16(39.0)		
High school and above	11(26.2)	8(19.5)		
Profession, n (%)			1.327	0.535^a^
Farmer	27(64.3)	24(58.5)		
Worker	6(14.3)	10(24.4)		
Other	9(21.4)	7(17.1)		
Way of living, n (%)			—	0.277^b^
With spouse and children	20(47.6)	24(58.5)		
With spouse	15(35.7)	8(19.5)		
With children	4(9.5)	7(17.1)		
Other	3(7.2)	2(4.9)		
The source of life, n (%)			0.311	0.856^a^
Pension	13(31.0)	14(34.1)		
Child support	20(47.6)	20(48.8)		
Subsistence allowance	9(21.4)	7(17.1)		
Average monthly household income (RMB), n (%)			1.832	0.608^a^
0~	11(26.2)	15(36.6)		
1000~	15(35.7)	11(26.8)		
2000~	5(11.9)	6(14.6)		
3000~	11(26.2)	9(22.0)		
Medical expenses, n (%)			—	0.559^b^
New rural cooperative medical system/urban medical insurance	33(78.6)	36(87.8)		
At own expense	6(14.3)	3(7.3)		
Public health care	3(7.1)	2(4.9)		
Hypertension, n (%)			0.057	0.811^a^
No	13(31.0)	12(29.3)		
Yes	29(69.0)	29(70.7)		
Diabetes, n (%)			0.051	0.821^a^
No	27(64.3)	25(61.0)		
Yes	15(35.7)	16(39.0)		
Heart disease, n (%)			0.192	0.661^a^
No	18(42.9)	20(48.8)		
Yes	24(57.1)	21(51.2)		
NIHSS score, n (%)			5.782	0.216^a^
0–1	16(38.1)	8(19.5)		
1–4	13(31.0)	11(26.8)		
5–15	9(21.4)	16(39.0)		
16–23	3(7.1)	3(7.3)		
Over 23	1(2.4)	3(7.3)		
Average length of stay (Mean ± SD)	14.55 ± 3.22	14.78 ± 3.56	-0.309	0.758^c^

Note: IG=intervention group; CG=control group; Heart disease includes coronary heart disease, arrhythmia, myocardial disease and rheumatic valvular heart disease. Worker includes manual laborers, factory workers, and service industry employees. SD=Standard deviation; ^a^: Chi-squared test; ^b^: Fisher’s exact test; ^c^: Independent samples t-test

#### Muscle strength

**Fig. 2:**
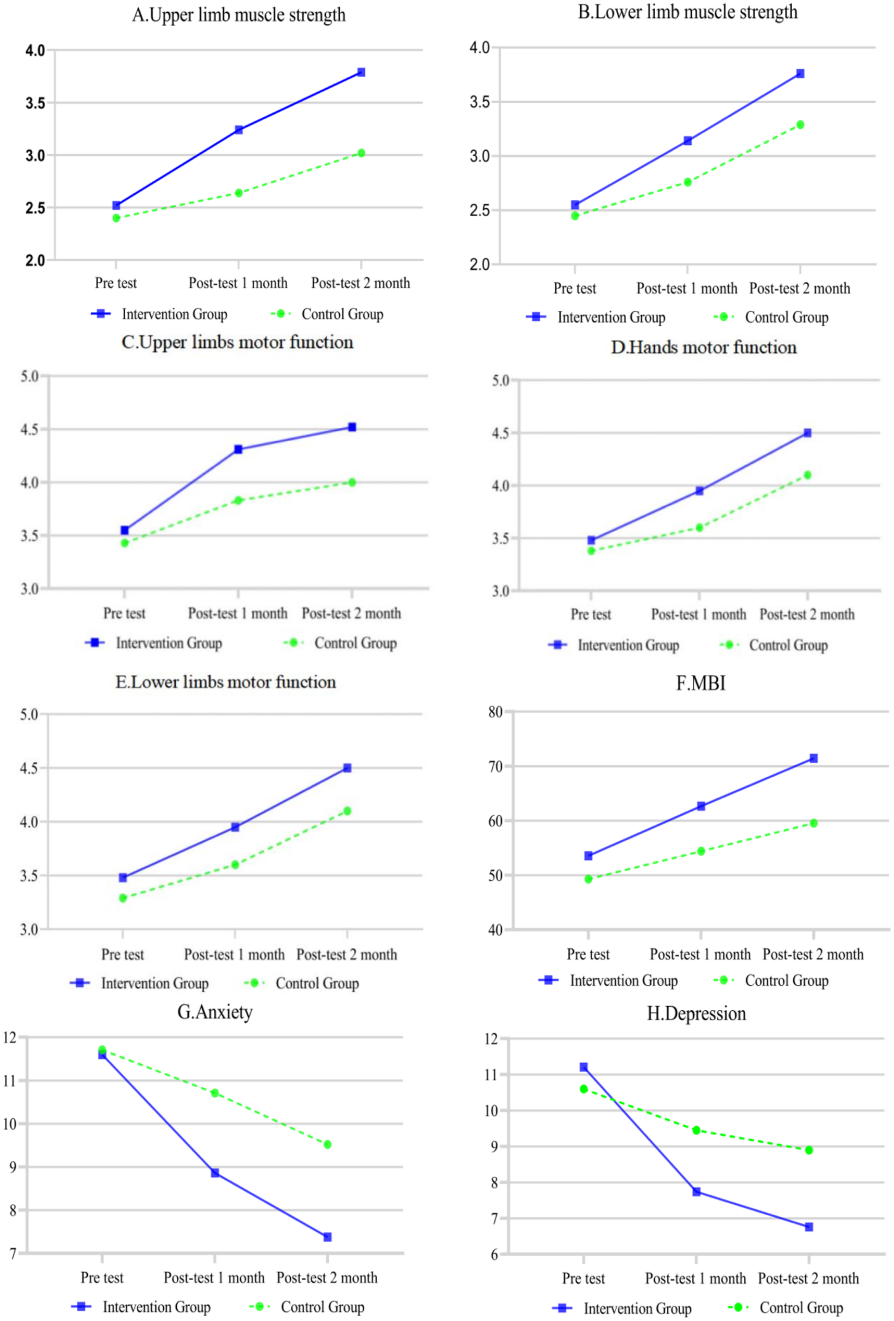
The Changes of Outcome Variables at Post-test 1 month (T1) and Post-test 2 months (T2): (**A**) Upper limb muscle strength, (**B**) Lower limb muscle strength, (**C**) Upper limbs motor function, (**D**) Hands motor function, (**E**) Lower limbs motor function, (**F**) MBI, (**G**) Anxiety, and (**H**) Depression

As shown in Table 3; Fig. 2A-B, the intervention group demonstrated significantly greater improvements in both upper and lower limb muscle strength compared with the control group at 1 month and 2 months post-intervention (all *p* < 0.001). At 2 months, the between-group differences were 0.77 (95% CI: 0.53–1.01) for upper limb and 0.47 (95% CI: 0.23–0.71) for lower limb, corresponding to large effect sizes (Cohen’s d = 1.43 and 0.86, respectively). Repeated-measures ANOVA revealed significant main effects of time (upper limb: F = 107.863, η²=0.571; lower limb: F = 186.343, η²=0.697; both *p* < 0.001) and group (upper limb: F = 21.667, η²=0.118; lower limb: F = 8.926, η²=0.052; both *p* < 0.01), as well as significant time-by-group interactions (upper limb: F = 14.782, η²=0.154; lower limb: F = 6.958, η²=0.079; both *p* < 0.05), indicating that the intervention group improved at a faster rate than the control group. Examination of the mean scores over time revealed progressive improvements at each consecutive assessment in the intervention group, whereas improvements in the control group were more gradual. This pattern was consistent with the significant time-by-group interactions observed in the repeated-measures ANOVA.

**Table 3:** Intergroup comparisons and repeated measures analysis of variance for muscle strength, motor function, and Modified Barthel Index (MBI) between the intervention group and the control group

Variables	Groups	Pre-test M ± SD	Post-test1 M ± SD	Post-test2 M ± SD	Repeated measurement ANOVA F(p), η_2_		
					Time	Group	Time×Group
Limb muscle strength							
Upper	IG(*n* = 42)	2.52 ± 0.51	3.24 ± 0.53	3.79 ± 0.42	F = 107.863 (*p* < 0.001)	F = 21.667 (*p* < 0.001)	F = 14.782 (*p* < 0.001)
	CG(*n* = 41)	2.40 ± 0.54	2.64 ± 0.73	3.02 ± 0.64	η²=0.571	η²=0.118	η²=0.154
	t(*p*)	1.039 (*p =* 0.302)	4.283 (*p* < 0.001)	6.447 (*p* < 0.001)			
	MD (95% CI)		0.60 (0.32, 0.88)	0.77 (0.53, 1.01)			
	Cohen’s d		0.94	1.43			
Lower	IG(*n* = 42)	2.55 ± 0.50	3.14 ± 0.57	3.76 ± 0.43	F = 186.343 (*p* < 0.001)	F = 8.926 (*p =* 0.004)	F = 6.958 (*p =* 0.001)
	CG(*n* = 41)	2.45 ± 0.50	2.76 ± 0.69	3.29 ± 0.64	η²=0.697	η²=0.052	η²=0.079
	t(*p*)	0.866 (*p =* 0.389)	2.762 (*p =* 0.007)	4.018 (*p* < 0.001)			
	MD (95% CI)		0.38 (0.10, 0.66)	0.47 (0.23, 0.71)			
	Cohen’s d		0.60	0.86			
Motor function							
Upper	IG(*n* = 42)	3.55 ± 0.67	4.31 ± 0.68	4.52 ± 0.63	F = 73.221 (*p* < 0.001)	F = 7.985 (*p =* 0.006)	F = 5.688 (*p =* 0.005)
	CG(*n* = 41)	3.43 ± 0.77	3.83 ± 0.66	4.00 ± 0.62	η²=0.475	η²=0.047	η²=0.066
	t(*p*)	0.756 (*p =* 0.452)	3.257 (*p =* 0.002)	3.814 (*p* < 0.001)			
	MD (95% CI)		0.48 (0.19, 0.77)	0.52 (0.25, 0.79)			
	Cohen’s d		0.72	0.83			
Motor function							
Hand	IG(*n* = 42)	3.48 ± 0.71	3.95 ± 0.66	4.50 ± 0.55	F = 119.887 (*p* < 0.001)	F = 4.281 (*p =* 0.042)	F = 4.354 (*p =* 0.016)
	CG(*n* = 41)	3.38 ± 0.73	3.60 ± 0.86	4.10 ± 0.66	η²=0.597	η²=0.026	η²=0.051
	t(*p*)	0.607 (*p =* 0.545)	2.139 (*p =* 0.036)	3.061 (*p =* 0.003)			
	MD (95% CI)		0.35 (0.01, 0.69)	0.40 (0.13, 0.67)			
	Cohen’s d		0.46	0.66			
Lower	IG(*n* = 42)	3.40 ± 0.66	4.05 ± 0.66	4.67 ± 0.53	F = 172.729 (*p* < 0.001)	F = 8.632 (*p =* 0.004)	F = 8.428 (*p* < 0.001)
	CG(*n* = 41)	3.29 ± 0.67	3.64 ± 0.76	4.10 ± 0.53	η²=0.681	η²=0.051	η²=0.094
	t(*p*)	0.816 (*p =* 0.417)	2.606 (*p =* 0.011)	4.950 (*p* < 0.001)			
	MD (95% CI)		0.41 (0.10, 0.72)	0.57 (0.34, 0.80)			
	Cohen’s d		0.58	1.08			
MBI							
	IG(*n* = 42)	53.55 ± 13.48	62.69 ± 11.01	71.45 ± 10.31	F = 135.243 (*p* < 0.001)	F = 11.671 (*p =* 0.001)	F = 9.911 (*p* < 0.001)
	CG(*n* = 41)	49.29 ± 12.14	54.40 ± 11.31	59.57 ± 9.91	η²=0.625	η²=0.067	η²=0.109
	t(*p*)	1.519 (*p =* 0.132)	3.401 (*p =* 0.001)	5.382 (*p* < 0.001)			
	MD (95% CI)		8.29 (3.41, 13.17)	11.88 (7.46, 16.30)			
	Cohen’s d		0.74	1.18			

Note: IG=intervention group; CG=control group; M ± SD = Mean ± standard deviation; MBI: Modified Barthel Index; ANOVA: Analysis of variance; η²: Eta squared (effect size); Cohen’s d: standardized effect size for independent samples t-test; CI; confidence interval

#### Motor function

Significant between-group differences in motor function were observed at both follow-up time points for the upper limbs, hands, and lower limbs (all *p* < 0.05; Table 3; Fig. 2C-E). At 2 months post-intervention, the intervention group demonstrated superior motor recovery, with mean differences of 0.52 (95% CI: 0.25–0.79) for upper limbs, 0.40 (95% CI: 0.13–0.67) for hands, and 0.57 (95% CI: 0.34–0.80) for lower limbs. These differences corresponded to moderate to large effect sizes (Cohen’s d = 0.83, 0.66, and 1.08, respectively). Repeated-measures ANOVA revealed significant main effects of time for all motor outcomes: upper limbs (F = 73.221, η²=0.475, *p* < 0.001), hands (F = 119.887, η²=0.597, *p* < 0.001), and lower limbs (F = 172.729, η²=0.681, *p* < 0.001). Significant main effects of group were also observed: upper limbs (F = 7.985, η²=0.047, *p* = 0.006), hands (F = 4.281, η²=0.026, *p* = 0.042), and lower limbs (F = 8.632, η²=0.051, *p* = 0.004). Furthermore, the time-by-group interactions were significant for all three measures: upper limbs (F = 5.688, η²=0.066, *p* = 0.005), hands (F = 4.354, η²=0.051, *p* = 0.016), and lower limbs (F = 8.428, η²=0.094, *p* < 0.001), confirming that the intervention group experienced a faster rate of functional recovery compared with the control group. Progressive improvements at each consecutive assessment were observed in the intervention group, whereas gains in the control group were more gradual, consistent with the significant time-by-group interactions confirming a faster recovery rate in the intervention group (Table 3).

#### MBI

The intervention group showed significantly higher Modified Barthel Index (MBI) scores than the control group at 1 month (MD = 8.29, 95% CI: 3.41–13.17, Cohen’s d = 0.74, *p* = 0.001) and 2 months (MD = 11.88, 95% CI: 7.46–16.30, Cohen’s d = 1.18, *p* < 0.001) post-intervention (Table 3; Fig. 2F). Repeated-measures ANOVA revealed significant main effects of time (F = 135.243, η²=0.625, *p* < 0.001) and group (F = 11.671, η²=0.067, *p* = 0.001), as well as a significant time-by-group interaction (F = 9.911, η²=0.109, *p* < 0.001). These results indicate that while both groups improved over time, the intervention group demonstrated a significantly steeper trajectory of recovery in daily living activities.

#### Anxiety and depression

As presented in Table 4; Fig. 2G-H, the intervention group exhibited significantly greater reductions in anxiety (GAD-7) and depression (PHQ-9) scores compared with the control group at both 1 month and 2 months post-intervention (all *p* < 0.01). At 2 months, between-group differences were − 2.14 (95% CI: -3.05 to -1.23) for anxiety and − 2.14 (95% CI: -2.95 to -1.33) for depression, with large effect sizes (Cohen’s d = 1.03 and 1.16, respectively). Repeated-measures ANOVA with Greenhouse-Geisser correction (applied due to violations of sphericity) showed significant main effects of time (anxiety: F = 56.321, η²=0.410, *p* < 0.001; depression: F = 66.300, η²=0.450, *p* < 0.001) and group (anxiety: F = 5.958, η²=0.035, *p* = 0.017; depression: F = 4.558, η²=0.027, *p* = 0.036). Significant time-by-group interactions were also observed (anxiety: F = 9.532, η²=0.105, *p* < 0.001; depression: F = 16.971, η²=0.173, *p* < 0.001), confirming that the intervention group experienced a faster rate of psychological improvement. Examination of the mean scores over time revealed progressive reductions in anxiety and depression at each consecutive assessment in the intervention group, whereas improvements in the control group were more gradual. This pattern was consistent with the significant time-by-group interactions (Table 4).

**Table 4:** Intergroup comparisons and repeated measures ANOVA for anxiety and depression between the intervention group and the control group

Variable	Group	Pre-test M ± SD	Post-test1 M ± SD	Post-test2 M ± SD	Repeated measurement ANOVA F(*p*), η²		
					Time	Group	Time×Group
Anxiety							
	IG(*n* = 42)	11.60 ± 3.68	8.86 ± 2.28	7.38 ± 1.53	F = 56.321^*^ (*p* < 0.001)	F = 5.958 (*p =* 0.017)	F = 9.532^*^ (*p* < 0.001)
	CG(*n* = 41)	11.71 ± 3.40	10.71 ± 3.12	9.52 ± 2.51	η²=0.410	η²=0.035	η²=0.105
	t(*p*)	-1.152 (*p =* 0.878)	-3.121 (*p =* 0.003)	-4.716 (*p* < 0.001)			
	MD (95% CI)		-1.85 (-3.05, -0.65)	-2.14 (-3.05, -1.23)			
	Cohen’s d		0.68	1.03			
Depression							
	IG(*n* = 42)	11.21 ± 3.63	7.74 ± 1.71	6.76 ± 1.21	F = 66.300^*^ (*p* < 0.001)	F = 4.558 (*p =* 0.036)	F = 16.971^*^ (*p* < 0.001)
	CG(*n* = 41)	10.60 ± 2.96	9.45 ± 2.83	8.90 ± 2.32	η²=0.450	η²=0.027	η²=0.173
	t(*p*)	0.863 (*p =* 0.394)	-3.358 (*p =* 0.001)	-5.301 (*p* < 0.001)			
	MD (95% CI)		-1.71 (-2.73, -0.69)	-2.14 (-2.95, -1.33)			
	Cohen’s d		0.73	1.16			

Note: IG=intervention group; CG=control group; M ± SD = Mean ± standard deviation; ANOVA: Analysis of variance; η²: Eta squared (effect size); Cohen’s d: standardized effect size for independent samples t-test; CI: confidence interval. *: Greenhouse-Geisser correction was applied due to violation of sphericity

## Discussion

CI is a leading cause of disability among elderly adults, profoundly affecting patients’ physical function, psychological well-being, and overall quality of life [46]. While previous studies have demonstrated the benefits of early rehabilitation in stroke populations [47, 48], the optimal structure and content of nursing-led interventions remain underexplored. The 5E rehabilitation model, originally developed for chronic disease management [30], offers a multi-dimensional framework that integrates psychological support, education, and functional training. In the present study, this model significantly improved muscle strength, motor function, daily living ability, and psychological outcomes in elderly patients with CI.These findings are consistent with those of Anjos et al. [49] and García-Pérez et al. [50], who reported that early, structured nursing interventions positively influenced motor and psychological recovery after stroke. However, unlike previous studies that focused primarily on the timing of intervention initiation [49] or single-component programs [50], the 5E model employed in this study is distinguished by its multi-component and nurse-coordinated nature. This may explain the relatively large effect sizes observed, particularly in psychological outcomes, where the combination of encouragement, education, and family involvement likely contributed to greater reductions in anxiety and depression than those reported in trials offering only psychological counseling.

The significant improvements in muscle strength, motor function, and daily living ability observed in the intervention group are consistent with the findings of Tanaka et al. [51], who reported that extended rehabilitation time was associated with greater functional gains. However, while Tanaka et al. attributed the improvements primarily to the duration of intervention, our results suggest that the structured and progressive nature of the 5E model may be equally important. The intervention began 24 h after stabilization, incorporating passive and active exercises (e.g., Bobath handshake, bridging, transfer training) in a stepwise manner tailored to individual capacity. This approach aligns with current neurorehabilitation principles emphasizing task-specific training and early mobilization [52]. This distinction has important clinical implications. Given that rehabilitation duration is often constrained by healthcare resources, optimizing the structure and progression of training may offer a more feasible pathway to enhancing functional outcomes.

The neurological deficits following CI result from irreversible neuronal damage and disruption of neural pathways, necessitating compensatory reorganization of the central nervous system for functional recovery [53]. This reorganization does not occur spontaneously but requires repetitive, task-specific training [54]. Our findings reinforce the importance of early, structured rehabilitation in facilitating this process. However, the optimal intensity and content of such training remain debated. While some studies have suggested that very early and high-intensity mobilization may not confer additional benefits and could even be harmful [55], our intervention adopted a progressive, patient-tailored approach that avoided overexertion while ensuring consistent stimulation. This may explain the favorable outcomes observed, with no intervention-related adverse events reported.

Maintaining exercise adherence after discharge remains a major challenge in stroke rehabilitation, with community-dwelling survivors often showing declines in physical activity over time [56]. The weekly telephone and WeChat follow-up in our study, combined with video-based exercise demonstrations, was associated with sustained engagement. This finding aligns with emerging evidence that technology-enhanced, remote monitoring can improve adherence and outcomes in stroke populations [57]. Unlike passive educational materials, the interactive nature of our follow-up —including review of exercise diaries and problem-solving discussions —may have strengthened patients’ self-efficacy and motivation. Future studies should explore the optimal intensity and duration of such remote support, as well as its cost-effectiveness compared with traditional in-person visits.

In addition to supporting physical adherence, the 5E model also directly targeted psychological well-being through its encouragement and family engagement components. Our results indicated significant differences in anxiety and depression scores between the intervention and control groups at both one month and two months post-intervention, with the intervention group showing greater improvement. The patient’s motor function and activities of daily living can influence their psychological status following CI, and in severe cases, patients may experience anxiety, depression, and other negative emotions [58]. This poor psychological status can, in turn, hinder the recovery process. This study illustrates that nursing staff should attend to the psychological health of elderly patients while focusing on the recovery of their physiological functions. By encouraging elderly patients to actively express their inner thoughts, sharing successful treatment cases, and facilitating peer support, this approach can enhance patients’ intrinsic motivation and reduce psychological resistance to behavioural change. Furthermore, family support plays a crucial role in alleviating anxiety and depression. The family communication skills module in this study promoted the dyadic relationship between patients and caregivers, consistent with communal coping models in chronic disease management [59, 60].

Notably, the 5E intervention was more intensive and comprehensive than the control group intervention, involving daily structured exercises, systematic psychological support, and weekly post-discharge follow-up. Therefore, the positive effects observed in the intervention group may reflect the combined intensity and multi-component nature of the 5E model, rather than any single element such as the timing of initiation or a specific exercise technique. This interpretation is consistent with evidence from populations with other cardiovascular conditions. Li et al. [61] demonstrated that a nurse-led multicomponent behavioural activation programme significantly improved health-related quality of life and disease-specific knowledge among patients with atrial fibrillation, with effects sustained at six months. Future research should consider using a dose-matched control group to isolate the specific contribution of the 5E framework from the effect of increased intervention intensity.

These findings suggest that the early rehabilitation nursing programme for elderly patients with CI is essential for supporting both physical and psychological function. The program equips nurses in hospital, community, and home settings with the knowledge to address patients’ physiological, psychological, and functional needs from the integrated perspectives of an educator, a supporter, and a coordinator, thereby promoting early recovery.

### Limitations

This study has several limitations. First, the single-center design and urban sample may limit the generalizability of the results, as participants likely had better access to technology and family support than their rural counterparts. Second, although allocation concealment was maintained and outcome assessors were blinded, the recruiting nurse became aware of group assignment after envelope opening; however, this risk is minimal as the nurse was not involved in outcome assessment or intervention delivery. Participants and providers could not be blinded, which may have introduced performance bias. Third, to prevent contamination, groups were placed on different hospital floors, which may have introduced environmental bias due to variations in staff or routine care. Fourth, the intervention was more intensive than the control, so the observed effects may reflect the combined intensity of the 5E model rather than its specific components; future studies should use a dose-matched control group. Fifth, the two-month follow-up precludes assessment of long-term sustainability. Finally, the trial was registered retrospectively, a methodological limitation in accordance with journal policies. Despite these limitations, this study provides preliminary evidence supporting the 5E model in elderly patients with CI, warranting validation in multi-center trials with larger, diverse samples and extended follow-up.

## Conclusions

The rehabilitation nursing program based on the 5E management model effectively improves upper and lower limb muscle strength, motor function, and activities of daily living, while alleviating anxiety and depression in elderly patients with cerebral infarction. These findings contribute to the evidence base for nursing care in this population and underscore the need for further multi-center evaluation to validate its clinical applicability. This approach may serve as a reference for continuous nursing and secondary prevention of stroke. Future multi-center studies with longer follow-up periods are warranted to evaluate the effectiveness of the 5E rehabilitation model in diverse settings and populations.

## Electronic supplementary material

Below is the link to the electronic supplementary material.

Supplementary Material 1

## Data Availability

The datasets will be available from the corresponding author upon reasonable request.
