# Supplementary material for: Effect of 5E rehabilitation management model on physical and psychological health of elderly patients with cerebral infarction: a randomized controlled trial
Source: BMC Nurs. 2026 Apr 24;25:592. doi: 10.1186/s12912-026-04671-3 (PMC13335186; doi:10.1186/s12912-026-04671-3)
Supplement: Supplementary file 1 — Supplementary Material 1 [file 12912_2026_4671_MOESM1_ESM.docx]

**Supplementary File 1: Detailed 5E Rehabilitation Intervention Protocol**

**1. Overview of the Intervention**

The 5E rehabilitation management model consists of five core components: Encouragement, Education, Exercise, Employment, and Evaluation. The intervention is delivered over a 2‑month period, beginning 24 hours after the patient’s vital signs and neurological symptoms have stabilized. It comprises a structured in‑hospital phase followed by post‑discharge telephone follow‑up. This document provides a session‑by‑session outline, educational materials, and fidelity monitoring tools to enable replication.

**2. Intervention Providers and Training**

Providers: Providers: Four trained research nurses.

Training: A 2‑week standardized training program conducted by a senior rehabilitation specialist and a neurologist, covering: (1)Theoretical basis of the 5E model. (2)Practical skills for passive/active rehabilitation exercises (e.g., Bobath handshake, bridging, transfer techniques). (3)Use of assessment tools: Manual Muscle Testing (MMT), Brunnstrom stages, Modified Barthel Index (MBI), PHQ‑9, GAD‑7.

(4)Communication techniques for psychological support and health education. (5)Role‑playing and supervised practice sessions.

Competency assessment: All providers passed a practical and written examination (>85% score) before trial commencement.

**3. Intervention Content by 5E Component**

3.1 Encouragement

Objective: Enhance motivation, reduce anxiety/depression, and foster a positive rehabilitation attitude.

| Second‑level Indicator | Detailed Description | Provider | Frequency |
| --- | --- | --- | --- |
| Encourage expression | Use open‑ended questions to explore patient’s feelings. Example: “How are you feeling about your recovery today?” Validate emotions and offer reassurance. | Nurse | Daily during hospitalisation |
| Case sharing | Share anonymised success stories of similar patients who improved through rehabilitation. Use photos/videos if available. | Nurse | Once during hospitalisation |
| Peer encouragement | Facilitate brief meetings between patients in the same ward (with similar functional levels) to share experiences and encourage each other. | Nurse | On admission |
| Family communication guidance | Teach family members basic communication skills: active listening, avoiding criticism, using positive reinforcement. | Nurse | Once a week during hospitalisation |
| Post‑discharge follow‑up | Weekly phone/WeChat calls: inquire about emotional state, provide encouragement, and address concerns. Use a standardised script (see Appendix A). | Nurse | Weekly for 2 months after discharge |

3.2 Education

Objective: Improve disease knowledge, self‑management skills, and treatment adherence.

| Second‑level Indicator | Detailed Description | Provider | Frequency |
| --- | --- | --- | --- |
| Collective health education | Group sessions (3–5 patients and their caregivers) covering: causes of cerebral infarction, risk factors, importance of rehabilitation, medication adherence, diet, and fall prevention. Use PowerPoint slides and printed leaflets. | Nurse | 3 sessions during hospitalisation (30–40 min each) |
| Individualized guidance | After each collective session, assess patient’s understanding via questions (e.g., “Can you tell me what foods are good for your recovery?”). Provide personalised clarification. | Nurse | Weekly during hospitalisation |
| Post‑discharge education | During follow‑up calls, reinforce key messages: medication schedule, healthy lifestyle, symptom recognition (e.g., warning signs of recurrent stroke). Use teach‑back method to confirm understanding. | Nurse | Weekly for 2 months after discharge |

Educational Materials (available upon request):

Leaflet and Slide deck : “Identification of Stroke Risk Factors” (Chinese version)

3.3 Exercise

Objective: Improve muscle strength, motor function, and mobility through progressive rehabilitation exercises.

| Second‑level Indicator | Detailed Description | Provider | Frequency |
| --- | --- | --- | --- |
| Turning over | Assist patient to change position every 2 hours to prevent pressure ulcers. Teach family members how to assist safely. | Nurse / Family | Every 2 hours during waking hours |
| Good limb position placement | Position patient in supine, healthy side‑lying, affected side‑lying, and bed‑sitting positions using pillows for support. Instruct on proper alignment. | Nurse | Every 2 hours |
| Passive movement of upper limbs | Perform slow, gentle movements of shoulder (flexion/extension, abduction/adduction), elbow, and wrist joints. Each movement 5–10 repetitions, within pain‑free range. | Nurse | Once daily (20–30 min) during hospitalisation |
| Passive hand movement | Move metacarpophalangeal and interphalangeal joints through full range. Include finger flexion/extension, thumb opposition. | Nurse | Once daily (part of upper limb session) |
| Passive movement of lower limbs | Perform hip/knee flexion/extension, ankle dorsiflexion/plantarflexion, and foot inversion/eversion. | Nurse | Once daily (20–30 min) |
| Active movement | Progressively increase active participation. Use Bobath handshake for upper limb training. For lower limbs: bridging, sitting up, sitting balance, standing balance, and walking training (with support as needed). Adjust difficulty based on patient’s ability. | Nurse | Once daily (20–30 min) during hospitalisation |
| Body position transfer | Train patient in bed mobility (rolling, moving up/down), transfers (lying to sitting, sitting to standing, bed to chair). Use step‑by‑step guidance and physical assistance as needed. | Nurse | Once daily during hospitalisation |
| Post‑discharge exercise guidance | During follow‑up calls, review exercise diary, provide feedback, and encourage continuation. Use video demonstrations (sent via WeChat) for correct technique. | Nurse | Weekly for 2 months after discharge |

Exercise Diary: Patients (or caregivers) record daily exercises (type, duration, any difficulties) in a provided logbook. Reviewed during follow‑up calls.

3.4 Employment

Objective: Enhance activities of daily living (ADL) and promote independence.

| Second‑level Indicator | Detailed Description | Provider | Frequency |
| --- | --- | --- | --- |
| Daily living ability training | Train patients in self‑care tasks: washing face, brushing teeth, eating, dressing, toileting, and grooming. Use adaptive equipment if needed. Break tasks into small steps; provide verbal cues and physical guidance. | Nurse | Once daily during hospitalisation |
| Post‑discharge ADL support | During follow‑up, ask about progress in daily activities, identify barriers, and suggest adaptations. Encourage family to allow patient to perform tasks independently as much as possible. | Nurse | Weekly for 2 months after discharge |

3.5 Evaluation

Objective: Assess baseline status, monitor progress, and adjust care plan.

| Second‑level Indicator | Detailed Description | Provider | Frequency |
| --- | --- | --- | --- |
| Admission evaluation | Collect demographic data, medical history, comorbidities, and social support information. | Nurse | On admission |
| Pre‑intervention evaluation | Assess muscle strength (MMT), motor function (Brunnstrom), ADL (MBI), anxiety (GAD‑7), and depression (PHQ‑9) 24 h after stabilisation. | Nurse / Assessor | Before intervention |
| Evaluation of intervention effect | Repeat all outcome measures at 1 month and 2 months post‑baseline. Use standardised protocols. | Blinded assessor | 1 month, 2 months |

**4. Post‑Discharge Follow‑Up Protocol**

Frequency: Weekly for 8 weeks.

Mode: Telephone or WeChat voice/video call, scheduled in advance.

Duration: 15–20 minutes.

Structure:

Greeting and rapport building.

Review of exercise diary (ask about types, frequency, difficulties).

Assessment of emotional state (using brief questions like “Have you felt anxious or depressed this week?”).

Reinforcement of educational messages (medication, diet, symptom recognition).

Problem‑solving for any barriers encountered.

Encouragement and goal setting for the next week.

Documentation: Each call is logged in a standardised form ( Appendix B).

**5. Fidelity Monitoring Tools**

5.1 Intervention Log (completed by nurse after each session)

| Date | Patient ID | Session Type (e.g., passive exercise) | Duration (min) | Activities Performed | Deviations from Protocol (if any) | Nurse Signature |
| --- | --- | --- | --- | --- | --- | --- |
|  |  |  |  |  |  |  |

5.2 Fidelity Checklist (used by supervisor during random observations)

| Item | Yes | No | Comments |
| --- | --- | --- | --- |
| Session started within scheduled time | ☐ | ☐ |  |
| Nurse followed the prescribed exercise sequence | ☐ | ☐ |  |
| Nurse provided appropriate verbal encouragement | ☐ | ☐ |  |
| Nurse used correct manual handling techniques | ☐ | ☐ |  |
| Patient safety maintained throughout | ☐ | ☐ |  |
| Session duration within 20–30 min range | ☐ | ☐ |  |
| Any adverse events? | ☐ | ☐ |  |
| Overall adherence rating (1–5) |  |  |  |

5.3 Post‑Discharge Call Log

| Patient ID | Date | Call Duration | Exercise Adherence (days/week) | Emotional Status (0–10 scale) | Issues Identified | Actions Taken | Nurse Initials |
| --- | --- | --- | --- | --- | --- | --- | --- |
|  |  |  |  |  |  |  |  |

**6. Appendices**

**Appendix A: Standardised script for post‑discharge follow‑up calls.**

Purpose of Call: To understand the patient's post-discharge rehabilitation status, provide emotional support, reinforce health education, address rehabilitation barriers, and encourage adherence to training.

Pre-Call Preparation: Prepare the patient's intervention records, exercise diary, and summary of the previous call.

Call Structure:

1.Opening and Rapport Building (1-2 minutes)

"Hello, Uncle/Auntie [Patient's Name]. This is Nurse [Nurse's Name] from [Hospital Name]. I'm calling today as scheduled to check on your recovery since discharge. This call will take about 15-20 minutes. Is this a convenient time for you to talk?"

If convenient, proceed. If not, schedule another time.

2.Inquiry About Recent Status (3-5 minutes)

"How have you been feeling this past week? Any physical discomfort?"

"How about your mood? Have you felt particularly anxious or down?" (If the emotional score is low, inquire further about the reasons.)

"How have your sleep and appetite been?"

3.Exercise Adherence Check (3-5 minutes)

"Did you manage to stick with your rehabilitation exercises this week? Approximately how many days, and for how long each day?"

"What kind of exercises did you mainly do? (e.g., passive upper limb movements, bridging exercises, standing training, etc.)"

"Did you encounter any difficulties? (e.g., pain, fatigue, lack of time, lack of motivation, etc.)"

"Would you mind telling me about your exercise diary? For example, what activities did you do yesterday?"

4.Health Education Reinforcement (3-5 minutes)

"Do you remember the key things we discussed paying special attention to after a stroke? (medication, diet, follow-up appointments, recognising warning signs)"

"Have you been taking your medication on time? Any forgotten or missed doses?"

"Are you paying attention to a low-salt, low-fat diet? Have you been eating plenty of fruits and vegetables recently?"

"If you experience sudden headache, worsening limb weakness, or slurred speech, remember to contact us immediately or go to the hospital. OK?"

5.Problem-Solving and Goal Setting (2-3 minutes)

"Regarding the difficulties you mentioned, we could try this..." (Provide specific suggestions, such as adjusting training time, simplifying movements, family assistance, etc.)

"Shall we aim for at least 20 minutes of training every day next week? You can set a small goal for yourself, like being able to stand independently for 1 minute."

6.Encouragement and Closing (1-2 minutes)

"You've done very well this week. Persisting with training is crucial for recovery. Keep up the good work!"

"Our next call will be at the same time next [day of the week]. I'll call you again. If you have any questions in the meantime, feel free to contact us via WeChat anytime."

"Wishing you a good week. Goodbye!"

Appendix B: Post‑discharge call log form (full page version).

Appendix B: Post-Discharge Call Log Form (Full Page Version)

| Patient ID | Call Date | Call Duration (minutes) | Exercise Adherence (days/week) | Emotional Status (0-10, 0=worst, 10=best) | Issues Identified | Actions Taken | Nurse Initials |
| --- | --- | --- | --- | --- | --- | --- | --- |
|  |  |  |  |  |  |  |  |
